# Hyperosmotic Shock Transiently Accelerates Constriction Rate in *Escherichia coli*

**DOI:** 10.3389/fmicb.2021.718600

**Published:** 2021-08-13

**Authors:** Jiawei Sun, Handuo Shi, Kerwyn Casey Huang

**Affiliations:** ^1^Department of Bioengineering, Stanford University, Stanford, CA, United States; ^2^Department of Microbiology and Immunology, Stanford University School of Medicine, Stanford, CA, United States; ^3^Chan Zuckerberg Biohub, San Francisco, CA, United States

**Keywords:** FtsZ, divisome, cytokinesis, sucrose, osmolarity, microfluidics

## Abstract

Bacterial cells in their natural environments encounter rapid and large changes in external osmolality. For instance, enteric bacteria such as *Escherichia coli* experience a rapid decrease when they exit from host intestines. Changes in osmolality alter the mechanical load on the cell envelope, and previous studies have shown that large osmotic shocks can slow down bacterial growth and impact cytoplasmic diffusion. However, it remains unclear how cells maintain envelope integrity and regulate envelope synthesis in response to osmotic shocks. In this study, we developed an agarose pad-based protocol to assay envelope stiffness by measuring population-averaged cell length before and after a hyperosmotic shock. Pad-based measurements exhibited an apparently larger length change compared with single-cell dynamics in a microfluidic device, which we found was quantitatively explained by a transient increase in division rate after the shock. Inhibiting cell division led to consistent measurements between agarose pad-based and microfluidic measurements. Directly after hyperosmotic shock, FtsZ concentration and Z-ring intensity increased, and the rate of septum constriction increased. These findings establish an agarose pad-based protocol for quantifying cell envelope stiffness, and demonstrate that mechanical perturbations can have profound effects on bacterial physiology.

## Introduction

The bacterial cytoplasm contains a dense combination of nucleic acids, proteins, and numerous metabolites that together generate a high internal osmotic pressure relative to the extracellular environment. These turgor pressures of ∼1–10 atm (Cayley et al., 2000; Deng et al., 2011) must be balanced to maintain cellular integrity. To counter this mechanical load, virtually all bacteria have a rigid, highly cross-linked peptidoglycan cell wall (Holtje, 1998) that can bear substantial stress (Yao et al., 1999). Bacterial cells typically encounter a wide range of environmental osmolalities, for example enteric bacteria rapidly transition between high and low osmolalities inside and outside the host, respectively (Gauthier, 2000). Rapid increases in external osmolality cause plasmolysis in which water exits the cytoplasm, leading to reduced stress on the cell envelope [membrane(s) and cell wall] and cytoplasm shrinkage (Cota-Robles, 1963; Pilizota and Shaevitz, 2013). During relatively small hyperosmotic shocks (≤100 mM of osmolyte), cell-wall insertion and elongation continue unaffected (Rojas et al., 2014), while larger hyperosmotic shocks inhibit growth (Walter, 1924; Christian and Scott, 1953; Scott, 1953; Christian, 1955) as well as intracellular diffusion and molecular mobility (van den Bogaart et al., 2007; Boersma et al., 2015). In general, whether intracellular processes are affected by osmolality changes has not been fully investigated, particularly how changes to mechanical load shape cellular structure and physiology.

Microfluidic devices are powerful tools for monitoring growth and division dynamics at single-cell resolution (Taniguchi et al., 2010; Wang et al., 2010; Campos et al., 2014; Taheri-Araghi et al., 2015; Camsund et al., 2020), particularly during acute environmental changes such as osmotic shocks (Pilizota and Shaevitz, 2012; Rojas et al., 2014, 2017; Zhou et al., 2015; Buda et al., 2016; Rojas and Huang, 2018; Yang et al., 2020). A salient example of the utility of microfluidics in bacterial cell biology is the recent discovery that the Gram-negative outer membrane (OM) can bear substantial mechanical stress (Rojas et al., 2018). In a flow cell, *Escherichia coli* cells were exposed to a large hyperosmotic shock, followed by detergent treatment that induced lysis. The large contraction upon lysis indicated that the stiffness of the OM is comparable to that of the cell wall (Rojas et al., 2018).

Although microfluidic devices can provide dynamical single-cell information and can be used to screen libraries using elaborate designs (Taniguchi et al., 2010; Camsund et al., 2020), cost and throughput is often limiting, with only one strain or species typically tested at a time. Moreover, microfluidic devices limit the movement of cells via rigid physical constraints and hence allow only cells within a particular size range to enter, which imposes additional mechanical stress and makes a single device incompatible with species across a wide range of shapes and sizes. The unintended selection of particular sizes in microfluidic devices also potentially introduces biases (Oliveira et al., 2020). The traditional alternative to microfluidic devices for single-cell imaging is agarose pads, which are versatile platforms that are easy to prepare and applicable for morphologically diverse species. Several recent studies have introduced high-throughput methods for rapidly imaging collections of strains on large-format agarose pads (Kuwada et al., 2015; Shi et al., 2017b), enabling screening of genome-scale libraries. However, it is difficult to track the effects of acute environmental transitions on single cells using agarose pads; instead one must rely on population averages measured pre- and post-transition. Moreover, in the time period required for pad preparation, physiological changes may have taken place that are not captured by snapshots. Thus, it remains unclear whether osmotic shock-related phenomena can be robustly probed in high-throughput on agarose pads.

As a critical part of the bacterial cell cycle, cell division is highly regulated. In bacteria, a ring of the conserved tubulin homolog FtsZ (the “Z-ring”) (Bi and Lutkenhaus, 1991; Dai and Lutkenhaus, 1991) assembles at mid-cell and initiates assembly of the divisome machinery (Goley et al., 2011; Barrows and Goley, 2021). After the Z-ring forms and recruits other division proteins, it progressively constricts the membrane (Osawa and Erickson, 2013) and directs synthesis of the septal cell envelope (Bisson-Filho et al., 2017; Yang et al., 2017) at a constant rate (Reshes et al., 2008b), resulting in growth of new hemispherical endcaps. FtsZ concentration has been linked to the proportion of dividing cells, which changes across nutrient conditions (Ward and Lutkenhaus, 1985; Weart and Levin, 2003; Weart et al., 2007; Hill et al., 2012). Regulation of FtsZ expression affects cell size homeostasis (Si et al., 2019) and FtsZ synthesis and degradation predict the timing of the first division in starved cells supplied with nutrient pulses (Sekar et al., 2018). While it remains unclear whether constriction of the bacterial inner membrane must fight against turgor pressure (Erickson, 2009, 2017), in fission yeast reduction of turgor pressure accelerates cell division (Basu et al., 2014; Chang, 2017). Taken together, bacterial cell division is a natural candidate for processes affected by environmental osmolality.

Here, we developed an agarose pad-based protocol for Gram-negative envelope stiffness measurements, and sought to establish that pad measurements could recapitulate previous findings regarding the stiffness of the OM. To our surprise, the population-averaged length of cells after hyperosmotic shock as observed on agarose pads was significantly smaller than expected based on microfluidic measurements. By tracking single-cell dynamics during an osmotic shock in a microfluidic flow cell, we discovered that the rate of cell division transiently increased after the shock, and the fraction of dividing cells was quantitatively consistent with the apparent decrease in cell length. Treatment with the division inhibitor aztreonam was sufficient to recover the same level of contraction as observed by tracking single cells, thereby enabling quantification of the effects of hyperosmotic shock and detergent treatment in high-throughput on agarose pads. Using a strain expressing a functional msfGFP-tagged FtsZ, we show that FtsZ concentration and Z-ring intensity increased rapidly upon osmotic shock, and that constriction dynamics were significantly accelerated relative to steady-state growth, supporting the hypothesis that the divisome can provide constrictive force that acts against turgor pressure.

## Results

### Apparent Length Contraction Due to Hyperosmotic Shock Is Much Larger on Agarose Pads Than in a Microfluidic Flow Cell

**FIGURE 1:**
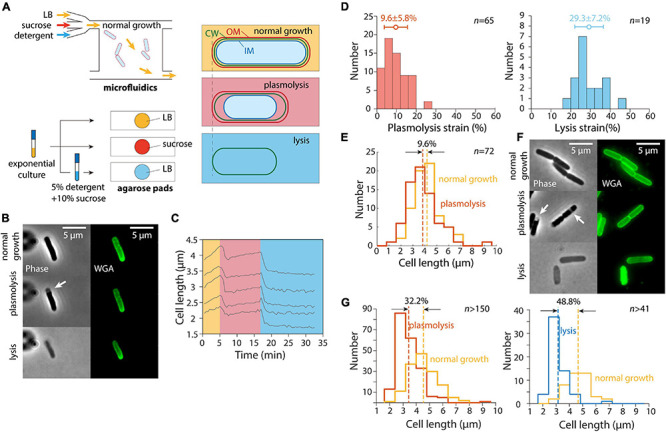
Population-averaged relative length change after hyperosmotic shock is greater on agarose pads than from single-cell tracking in microfluidic devices. **(A)** Schematic of microfluidic (top) and agarose pad-based (bottom) measurements of length changes (right) due to hyperosmotic shock (plasmolysis) and detergent-mediated lysis. **(B)** Relative to normal growth (top), cells exposed to a hyperosmotic shock in a microfluidic flow cell (middle) decreased in length and exhibited plasmolysis (white arrow). Subsequent lysis due to detergent treatment (bottom) resulted in further shrinkage. Shown are phase contrast images (left) and fluorescence from the wall label WGA-AF488 (right). **(C)** Quantification of cell length dynamics for 5 representative cells for the experiment in panel **B**. Cell elongation was inhibited after the shock. **(D)** Quantification of the mechanical strain (extension relative to the smaller state) in cell wall length due to hyperosmotic shock (left) and detergent treatment (right). The additional strain revealed by detergent treatment shows that the stiffness of the outer membrane is comparable to that of the cell wall (Rojas et al., 2018). **(E)** Population-averaged cell wall length of the same set of cells in a microfluidic device before and after hyperosmotic shock yielded a similar estimate of plasmolysis strain as in panel **D**. **(F)** Cells imaged during normal growth in a test tube (top), on agarose pads with 20% sucrose to cause a hyperosmotic shock (middle, white arrows indicate plasmolysis bays), and after detergent treatment to cause lysis (bottom). **(G)** The relative change in length of cells on an agarose pad before and after a hyperosmotic shock (left) was substantially greater than the relative length change in a microfluidic device shown in panel **D**, as was the relative length change after lysis (right).

In a previous study (Rojas et al., 2018), we exposed *E. coli* cells as well as other Gram-negative bacteria to a large hyperosmotic shock in a microfluidic flow cell to remove turgor pressure (Figure 1A). The plasmolyzed, shrunken cells were then exposed to either EDTA (to remove lipopolysaccharides from the OM) or detergent (to remove the OM altogether); both treatments caused the length of the cell wall to decrease even further (Rojas et al., 2018), indicating that the OM was exerting substantial stretching forces on the cell wall in the plasmolyzed state. We carried out similar experiments and observed similar cell wall mechanical strains (normalized length changes relative to the unextended state) using the wall dye wheat germ agglutinin conjugated to AlexaFluor 488 (WGA-AF488) (Ursell et al., 2014) upon hyperosmotic shock and detergent treatment (Figures 1B,C); unshocked cell walls were extended by 9.6% relative to shocked cells, with a larger contraction upon lysis (Figure 1D).

While tracking of cells within the microfluidic device allowed us to measure the relative length change of each cell throughout the hyperosmotic shock and subsequent OM destabilization, we found that the plasmolysis strain could be accurately quantified based on the population-averaged length before and after each treatment (Figure 1E). Moreover, cell growth was inhibited for at least 5 min after the hyperosmotic shock, with most non-dividing cells maintaining a stable length (Figure 1C). Thus, we hypothesized that we could take advantage of traditional agarose pad-based imaging to quantify OM stiffness in high throughput (Figure 1A).

To test whether agarose pad measurements would recapitulate our microfluidics-based measurements of length changes due to hyperosmotic shock, we exposed an exponentially growing *E. coli* culture in LB to the same hyperosmotic shock as in our microfluidics experiments. To perform the shock as quickly as possible, we directly spotted cells onto agarose pads containing LB+20% sucrose to induce a large (∼0.5 M) osmotic shock, and imaged the cells as quickly as possible (Figures 1A,F). To our surprise, the mean length strain was 32.2% (Figure 1G), far larger than for cells in the microfluidic device (Figures 1D,E).

Next, we diluted exponentially growing cells into a concentrated solution of detergent and sucrose (see section “Materials and Methods”) to induce cell lysis. The lysed cells were spotted onto agarose pads with LB for imaging after 20 min of detergent treatment. The decrease in cell length after lysis (Figure 1G) was also much larger than expected based on our microfluidic measurements (Figure 1D). Thus, we conclude that despite the seemingly simple nature of our length measurements, there was an unidentified factor that was affecting cell length differentially in the microfluidic device and on agarose pads.

### Osmotic Shock Transiently Increases the Rate of Cell Division

**FIGURE 2:**
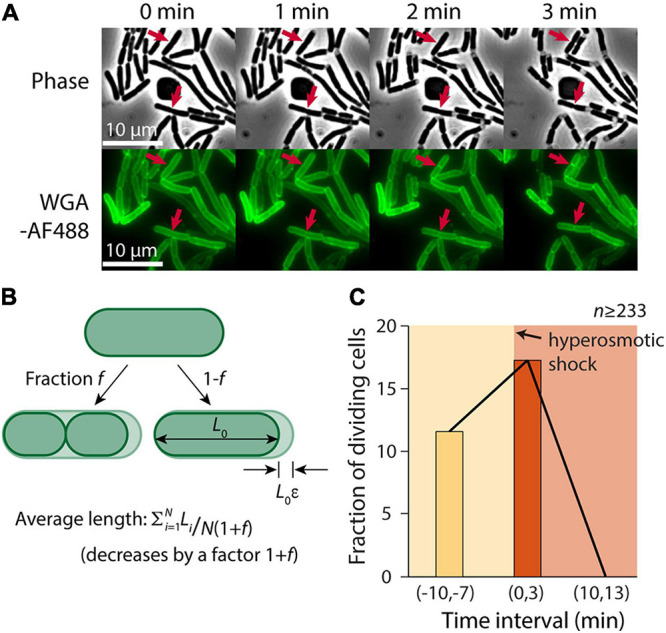
A transient burst of cell division follows hyperosmotic shock. **(A)** In the 3 min after exposure to a hyperosmotic shock, a fraction of cells continued to divide (red arrows indicate a few examples) despite growth largely halting (Figure 1C). Shown is a time-lapse in phase contrast (top) and fluorescence of the wall label WGA-AF488 (bottom). **(B)** If a fraction *f* of cells divide during the interval between the hyperosmotic shock and imaging on an agarose pad, the average length of cells post-shock will decrease by a factor (1+*f*), leading to a predicted increase in the plasmolysis strain by (1+*f*). **(C)** The fraction of dividing cells (see section “Materials and Methods”) increased from 11.6% pre-shock to 17.3% in the 3 min following the shock. By 10 min after the shock, division had halted completely.

Despite the slowing of elongation after the hyperosmotic shock (Figure 1C), we noted that some cells continued to divide (Figure 2A). Thus, we hypothesized that one cause of the discrepancy between our microfluidic and agarose pad measurements was the subset of cells that completed constriction in the time interval between the shock and the time of image acquisition, resulting in a decrease in the population-averaged length post-shock. For a population of cells with average length *L* during exponential growth that is extended by a factor ε relative to its shocked length *L*_0_, *L* = *L*_0_(1+ε). If the fraction of cells that divide between the time of the shock and agarose pad imaging is *f*, the total number of cells increases by the factor (1+*f*). Therefore, the mean length of shocked cells decreases by (1+*f*), yielding an apparent extension during exponential growth relative to shocked cells of (1+ε)(1+*f*) – 1 = ε+*f*+ε*f* (Figure 2B). Thus, we hypothesized that the fraction of cells that were dividing after the shock was approximately 20%, based on the difference in the apparent extension on pads and in the microfluidic device.

To test this hypothesis, we manually identified cells that divided in a 3-min time interval before and after the hyperosmotic shock using microfluidics. Because it is difficult to pinpoint the time of division initiation due to the diffraction limit of light microscopy, we used WGA-AF488 to label the cell wall and identified cells that formed a clear septum during the 3-min interval (see section “Materials and Methods”). The fraction of dividing cells pre-shock was 11.6% per 3 min (Figure 2C), consistent with our measured doubling time of ∼23 min (Supplementary Figure 1). In the 3 min after the shock, the dividing fraction increased to 17.3% (Figure 2B), in reasonable agreement with our prediction. Division halted completely 10 min after the shock, likely due to the lack of growth (Figure 2C). Thus, we conclude that cell division was a major factor in the discrepancy between contraction estimates.

### Inhibition of Cell Division Restores the Same Length Contraction After Hyperosmotic Shock as in Microfluidics

**FIGURE 3:**
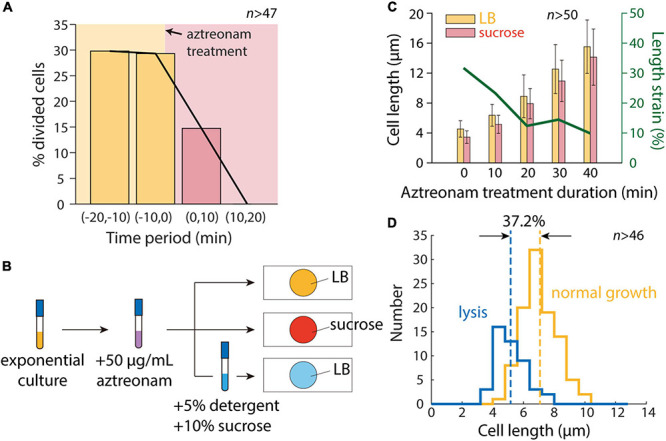
Inhibition of cell division allows for accurate measurement of length contraction after hyperosmotic shock. **(A)** During time-lapse imaging of *E. coli* cells treated with 50 μg/mL aztreonam, cell division was fully inhibited within 20 min. **(B)** Schematic of modified protocol for agarose pad-based measurements of cell envelope stiffness, in which cells are treated with 50 μg/mL aztreonam for various amounts of time before imaging on agarose pads. **(C)** Mean length of cells grown in a test tube with 50 μg/mL aztreonam increased with the duration of treatment, due to the inhibition of cell division. After a hyperosmotic shock on an agarose pad with LB+20% sucrose, the measured length strain gradually decreased with the duration of treatment and plateaued at ∼10%, consistent with microfluidic device measurements (Figure 1D). **(D)** After detergent treatment, the population-averaged length of aztreonam-treated cells was lower by an amount corresponding to a strain of 37.2%, consistent with microfluidic device measurements (Figure 1D).

To determine whether cell division was the sole factor distinguishing our microfluidic and agarose pad measurements, we treated cells with the beta-lactam aztreonam, which inhibits the division-specific transpeptidase PBP3 (Spratt, 1975). We exposed cells to 50 μg/mL aztreonam in a microfluidic flow cell to determine the time scale of complete division inhibition, and found that division completely halted within 20 min (Figure 3A). We then exposed cells in a test tube to aztreonam for varying amounts of time before hyperosmotic shock (Figure 3B), and as expected mean length increased monotonically with the duration of treatment (Figure 3C). Moreover, the degree of contraction after hyperosmotic shock relative to initial length decreased with the duration of treatment, plateauing after 20 min (Figure 3C), consistent with the time scale of complete division inhibition (Figure 3A).

We then exposed cells treated for 20 min with aztreonam to a solution of sucrose and detergent. The vast majority of cells lysed (>90%), and the mean length decreased such that unshocked cells were extended by 37.2% relative to the lysed cell wall length (Figure 3D). This value is reasonably consistent with the distribution of extensions of single cells in a microfluidic device (Figure 1D). We also treated cells with detergent in the absence of a hyperosmotic shock, surmising that the removal of the OM should remove both the membranes and turgor pressure, and thereby allow the cell wall to relax to its unstretched state. To our surprise, most cells (>95%) failed to lyse, suggesting that cells are more susceptible to detergent while plasmolyzed.

Thus, we conclude that the transient burst of cell division after hyperosmotic shock is responsible for the large decrease in population-averaged cell length, and that division inhibition is sufficient to enable pad-based measurements of cell envelope mechanical properties.

### Septum Formation Time Is Largely Constant Across Mutants With Variable Cell Width

**FIGURE 4:**
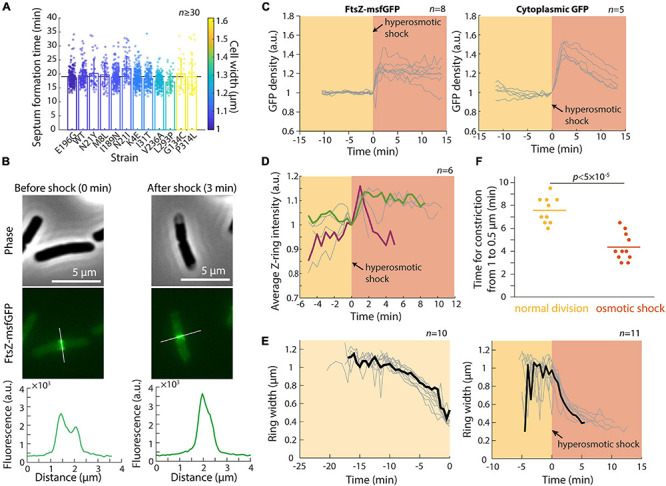
Hyperomostic shock leads to increased FtsZ concentration and ring intensity and faster constriction. **(A)** Septum formation time (see section “Materials and Methods”) for *mreB* mutants of various cell widths was approximately constant as a function of mean cell width. **(B)** Phase-constrast (top) and epifluorescence (middle) images of *E. coli* cells expressing FtsZ-msfGFP, before (left) and 3 min after (right) a hyperosmotic shock. Fluorescence profiles along the cell contour show that Z-ring intensity increased immediately after the shock (bottom). **(C)** The concentration of FtsZ-msfGFP (left) and cytoplasmic GFP (right) increased directly after the hyperosmotic shock, consistent with the decrease in cell volume (Figure 1B). **(D)** Z-ring intensity increased after the shock. Shown are 6 representative cells; the cell colored in green maintained high Z-ring intensity for 10 min after the shock, while Z-ring intensity in the maroon cell increased and then decreased for the ensuing 4 min leading up to the completion of cell division. **(E)** Z-rings constricted more rapidly after hyperosmotic shock (right) than during normal growth (left). The black curves are representative cells in each category. **(F)** The time required for Z-ring constriction was substantially shorter after hyperosmotic shock.

In a previous study, we showed that the concentration of FtsZ is largely constant across a set of MreB mutants with various cell widths and volumes (Shi et al., 2017a). However, the Z-ring in wider mutants was both broader and more intense (Shi et al., 2017a), suggesting that Z-ring assembly is width-dependent, which may lead to different dynamics during septum formation. To further probe the connection between cell width and septum formation, we selected a set of MreB mutants with different cell widths but similar growth rates in exponential phase (Shi et al., 2017a), and performed time-lapse imaging to determine the duration of septum formation in each strain (see section “Materials and Methods”). In these strains, mean cell width varied from 1 μm to 1.6 μm, corresponding to a ∼2.5-fold difference in septum areas. Nonetheless, all strains completed septum formation in ∼19 min, regardless of their width (Figure 4A; *r* = −0.13, *p* = 0.68, two-sided Student’s *t*-test). Thus, the broader and more intense Z-rings in wider cells potentially boost the rate of septum formation and allow cells to synthesize a larger septum within the same duration.

### FtsZ Concentrates at Midcell After Hyperosmotic Shock

To probe the mechanism underlying the transient increase in division after hyperosmotic shock, we performed time-lapse imaging in a microfluidic flow cell using an *E. coli* MG1655 strain expressing *ftsZ* fused to *msfGFP* as the sole copy of *ftsZ* (Figure 4B). This strain exhibits approximately normal growth (Moore et al., 2017) although the fusion does affect cell length by altering FtsZ GTPase activity (Yang et al., 2017). We first quantified the total amount and concentration of FtsZ within each cell (see section “Materials and Methods”). FtsZ concentration (total amount normalized by cell volume) increased directly after hyperosmotic shock (Figure 4C), as expected based on cytoplasmic shrinkage. A similar concentration increase was observed for another divisome protein, ZapA (Buss et al., 2013), in a strain expressing GFP-ZapA (Supplementary Figure 2), as well as in a control strain expressing cytoplasmic GFP (Figure 4C). In all cases, the increase was larger than the normalized length decrease of the cell wall, due to plasmolysis collapsing the cytoplasm away from the cell wall (Figure 4B). In addition to FtsZ and ZapA, other divisome proteins likely increase rapidly in concentration given the short time scale of cytoplasmic contraction.

To address whether the increase in FtsZ concentration is associated with accelerated constriction, we quantified the intensity and width of the Z-ring (Shi et al., 2017a). Strikingly, Z-ring intensity increased almost immediately after the shock (Figure 4D). Moreover, Z-ring constriction proceeded at a much faster pace in shocked compared with non-shocked cells (Figures 4E,F), despite the lack of overall volume expansion (Figure 1C). This accelerated Z-ring constriction largely explains the increase in the fraction of cells that complete constriction shortly after osmotic shock (Figure 2C). It is unclear whether the rate of division initiation also increased; regardless, initiation rate is unlikely to be the only cause of increased cell division after shock since septum formation typically requires ∼10–15 min during normal growth. Together, these results support the hypothesis that the enhanced rate of cell division post-hyperosmotic shock is due at least in part to the higher intensity of the Z-ring and faster rate of constriction.

## Discussion

In this study, we developed an agarose pad-based measurement protocol to quantify the stiffness of the OM from cell length measurements after hyperosmotic shock and lysis. Our measurements of length strain after plasmolysis were inconsistent between agarose pads and microfluidic devices (Figure 1), which was quantitatively explained by the increase in cell division directly after hyperosmotic shock (Figures 2–4). Treatment with the division inhibitor aztreonam restored the expected degree of length strain (Figure 3C), and thus enables characterization of osmotic shock-induced morphological changes and OM stiffness on agarose pads (Figure 3D). This strategy provides a simpler and more versatile methodology than microfluidics, especially for screening a diverse set of species or mutants. Using single-cell tracking, we discovered that FtsZ (Figure 4C) and ZapA (Supplementary Figure 2) concentration and Z-ring intensity (Figure 4D) increased and Z-ring constriction accelerated (Figures 4E,F) directly after a hyperosmotic shock, providing mechanistic hypotheses for the increased rate of division during plasmolysis. Division may also be affected by the likely increase in the concentration of cell-envelope precursors after the shock.

Our findings suggest that progression of bacterial cell division is coupled to environmental osmolality. The observation that the Z-ring constricts more quickly in the absence of turgor suggests that the divisome exerts constrictive forces on the envelope. During a hyperosmotic shock, both the decrease in turgor and increase in FtsZ concentration may contribute to the acceleration of constriction; it is difficult to decouple the two factors, since the cytoplasmic shrinkage that accompanies the reduced stress on the cell envelope inevitably concentrates FtsZ and presumably all other divisome components. Given the immediate response of cells to osmotic shock, the association between division and osmolarity points to possible ecological implications wherein transitions to a high osmotic environment that limits growth will result in proliferation into a larger population of cells that increases survival odds, reminiscent of the reductive divisions that take place when cells enter stationary phase (Sutterlin et al., 2016). Perhaps turgor decreases along with stationary-phase entry to facilitate division, or in other environmental transitions in which an increase in cell number would be beneficial. In our experiments, division halted almost completely after the initial boost induced by the shock despite the higher concentration of FtsZ relative to normal growth (Figure 2C), potentially due to other limiting factors that prevented growth (Figure 1C). It remains unclear how the activities of FtsZ and other divisome components are generally affected by osmolarity or intracellular crowding.

Our finding that cell division is affected by osmolarity suggests that environmental perturbations such as osmotic shocks may generally induce widespread cellular responses. Indeed, *E. coli* cells were found to respond in a coupled manner to heat and oxygen shock (Tagkopoulos et al., 2008), likely reflecting the correlation between high temperature and anoxia in human hosts. Certain antibiotics also induce acid stress (Mitosch et al., 2017) or heat-shock response pathways concurrently (Evans et al., 2019). In addition to the divisome, osmolality may affect other cellular structures such as the cytoskeletal filament MreB (Szatmári et al., 2020) and the nucleoid (Finan and Guilak, 2010; Cagliero and Jin, 2013; Wu et al., 2019; Yang et al., 2020), both of which could have widespread downstream physiological consequences. Our findings confirm that agarose pads can be used as a high-throughput platform for bacterial cell mechanics measurements, although microfluidics provides a critical ground truth in which the fate of single cells can be tracked. Thus, rapid screening of large libraries of mutants (Baba et al., 2006; Peters et al., 2016) or species should be straightforward as long as cell division can be inhibited, which should help to uncover the molecular basis of envelope stiffness and understand the role of physical forces in shaping cellular structures and bacterial physiology.

## Materials and Methods

### Strain Culturing

*Escherichia coli* cells were grown overnight in LB at 37°C and used to inoculate a test tube with fresh LB. Strains used in this study are listed in Supplementary Table 1.

### WGA Staining of the Cell Wall

Cell wall was labeled with wheat germ agglutinin (WGA) conjugated to AlexaFluor-488 (AF488, Invitrogen W11261). WGA-AF488 was added to exponential phase cells at a final concentration of 25 μg/mL and incubated in dark conditions with shaking at 37°C for at least 2.5 h prior to imaging.

### Hyperosmotic Shock Application on Agarose Pads

To apply a hyperosmotic shock on an agarose pad, cells were first cultured in liquid LB into steady-state exponential growth using the following serial dilution protocol: starting with a 1:200 dilution from an overnight saturated culture, 1:10 dilutions were performed after 60 and 150 min. After the second dilution, ∼1 μL of liquid culture was spotted onto a 1% agarose pad with LB+20% sucrose, leading to a hyperosmotic shock.

### Detergent Treatment for Agarose-Pad Measurements

Exponentially growing cells were exposed to a lysis solution of N-lauroylsarcosine sodium salt (detergent) and sucrose dissolved in 1X PBS. By directly diluting cultures into the lysis solution at a 1:1 ratio, the final concentration of detergent and sucrose was 5% and 10%, respectively. Sucrose was added to promote lysis, recapitulating the perturbation in a microfluidic device in which cells were plasmolyzed by a hyperosmotic shock before being exposed to detergent.

### Single-Cell Imaging

One microliter of cells was spotted onto a 1% agarose pad with the appropriate medium, and imaged on a Nikon Eclipse Ti-E inverted fluorescence microscope with a 100X (NA 1.40) oil-immersion objective (Nikon Instruments). For microfluidic experiments, cells were loaded into B04A (CellASIC) microfluidic plates following previous protocols (Rojas et al., 2018). Phase-contrast and epifluorescence images were collected on a DU885 electron-multiplying CCD camera (Andor Technology) or a Neo sCMOS camera (Andor Technology) using μManager v. 1.4 (Edelstein et al., 2010). Cells were maintained at 37°C during imaging with an active-control environmental chamber (Haison Technology).

### Identifying Cell Division Events From WGA Signal

In a microfluidic flow cell, cell division events were identified based on the clear formation of a septum in the WGA-AF488 signal. Although septum formation does not represent the end of cell division, the septum can be easily identified visually or from a peak in the fluorescence profile along the long axis of the cell. The fraction of dividing cells was computed based on a combination of visual inspection and fluorescence profile examination, with constriction dynamics in the phase channel serving as further confirmation.

### Image Analysis

The MATLAB (MathWorks, Natick, MA, United States) image processing code *Morphometrics* (Ursell et al., 2017) was used to segment cells and to identify cell outlines from phase-contrast and epifluorescence microscopy images. A local coordinate system was generated for each cell outline using a method adapted from *MicrobeTracker* (Sliusarenko et al., 2011). Cell length was calculated as the length of the midline from pole to pole. See figure legends for the number of cells analyzed (*n*) and error bar definitions.

### Quantification of FtsZ Fluorescence

Total FtsZ fluorescence was calculated by integrating fluorescence values within the cell contour after background subtraction, and FtsZ concentration was computed as the total fluorescence divided by cross-sectional area. The fluorescence profile along the cell contour had low values at the cell poles and high values at mid-cell, corresponding to the Z-ring. Z-ring intensity was calculated by averaging the fluorescence intensity within the Z-ring (Shi et al., 2017a).

### Tracking the Dynamics of Septum Formation

Assuming cells incorporate new septum area at a constant rate *k* and the shape of the cell poles is hemispherical, the septum area *A*(*t*) changes as a function of time according to $\frac{dA\left(t\right)}{dt}=k$, and A(t)=4πw021-(w(t)/w0)2, where, *w*_0_ is initial septum width (i.e., cell width) and *w*(*t*) is the current septum width (Reshes et al., 2008a). Integration yields

t=4πw02k1-(w(t)/w0)2,

where *t* = 0 corresponds to division onset. Through linear fitting of experimental measurements of $\sqrt{1-{\left(w\left(t\right)/w_{0}\right)}^{2}}$ as a function of *t*, we obtained the time required for septum formation, T=4πw02k. To reduce the error in estimating septum width, only time points with 0.5*w*_0_ ≤ *w*(*t*) ≤ 0.9*w*_0_ were used for the fit.

## Data Availability Statement

The original contributions presented in the study are included in the article/Supplementary Material, further inquiries can be directed to the corresponding author.

## Author Contributions

JS, HS, and KCH designed the research, wrote the manuscript, and analyzedthe data. JS and HS performed the research. All authors contributed to the article and approved the submitted version.

## Conflict of Interest

The authors declare that the research was conducted in the absence of any commercial or financial relationships that could be construed as a potential conflict of interest.

## Publisher’s Note

All claims expressed in this article are solely those of the authors and do not necessarily represent those of their affiliated organizations, or those of the publisher, the editors and the reviewers. Any product that may be evaluated in this article, or claim that may be made by its manufacturer, is not guaranteed or endorsed by the publisher.

## Supplementary Material

Supplementary Figure 1 The distribution of steady-state growth rates is similar in microfluidic devices and on agarose pads.

Supplementary Figure 2 GFP-ZapA concentration increases directly after a hyperosmotic shock. This increase was consistent with the decrease in cytoplasmic volume (Figure 1B) and the increases in FtsZ-msfGFP and cytoplasmic GFP (Figure 4C).

Supplementary Table 1 Strains used in this study.
