# Supplementary material for: Hyperosmotic Shock Transiently Accelerates Constriction Rate in Escherichia coli
Source: Front Microbiol. 2021 Aug 13;12:718600. doi: 10.3389/fmicb.2021.718600 (PMC8418109; doi:10.3389/fmicb.2021.718600)
Supplement: Supplementary Figure 1 — The distribution of steady-state growth rates is similar in microfluidic devices and on agarose pads. [file Data_Sheet_1.docx]

**Supplemental Figures**

**
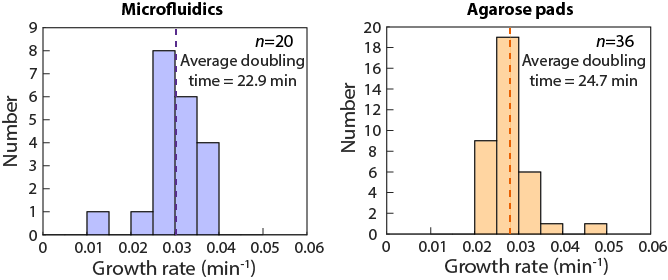
**

**Supplemental Figure 1: The distribution of steady-state growth rates is similar in microfluidic devices and on agarose pads.**

**
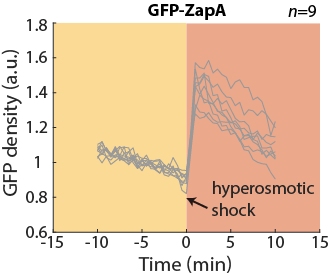
**

**Supplemental Figure 2: GFP-ZapA concentration increases directly after a hyperosmotic shock.** This increase was consistent with the decrease in cytoplasmic volume (Figure 1B) and the increases in FtsZ-msfGFP and cytoplasmic GFP (Figure 4C).

**Supplemental Table**

**Table S1: Strains used in this study.**

| Strain | Genotype | Source/reference |
| --- | --- | --- |
| *E. coli* MG1655 | F^-^, lambda^-^, *rph-1* | CGSC #6300 |
| KC376 | MG1655 *ftsZ*::*ftsZ-msfGFP* | (Moore et al., 2017) |
| KC1291 | *imp4213*, pCA-GFP-ZapA | (Yang et al., 2017) |
| RDM893 | MG1655 ∆*mreB* pRMmreBCD | (Shi et al., 2017a) |
| MreB-E196G | RDM893 MreB-E196G | (Shi et al., 2017a) |
| MreB-N21Y | RDM893 MreB-N21Y | (Shi et al., 2017a) |
| MreB-M8L | RDM893 MreB-M8L | (Shi et al., 2017a) |
| MreB-I189N | RDM893 MreB-I189N | (Shi et al., 2017a) |
| MreB-N21I | RDM893 MreB-N21I | (Shi et al., 2017a) |
| MreB-K4E | RDM893 MreB-K4E | (Shi et al., 2017a) |
| MreB-I31T | RDM893 MreB-I31T | (Shi et al., 2017a) |
| MreB-V236A | RDM893 MreB-V236A | (Shi et al., 2017a) |
| MreB-L293P | RDM893 MreB-L293P | (Shi et al., 2017a) |
| MreB-G134C | RDM893 MreB-G134C | (Shi et al., 2017a) |
| MreB-P314L | RDM893 MreB-P314L | (Shi et al., 2017a) |
